# Estimated GFR versus creatinine clearance for evaluation of recovery from acute kidney injury

**DOI:** 10.1186/cc13575

**Published:** 2014-03-17

**Authors:** P Timmermans, J Gunst, G Van den Berghe, M Schetz

**Affiliations:** 1 KU Leuven University Hospital, Leuven, Belgium

## Introduction

The aim of this study is to quantify the impact of using eGFR instead of measured creatinine clearance (Clcr) on the evaluation of recovery from acute kidney injury (AKI).

## Methods

From a large RCT's database [1] we excluded patients with end-stage renal disease and kidney transplants. In the remaining 4,560 patients, 1,296 (28%) developed AKI (KDIGO criteria). After exclusion of ICU nonsurvivors (*n *= 229), patients on dialysis at ICU discharge (*n *= 77) and patients for whom Clcr on the last day of ICU was not available (*n *= 206), 784 patients were included in this analysis. We compared eGFR (MDRD equation) with measured Clcr (based on 24-hour urine collection and corrected for BSA) at ICU discharge for patient groups with different ICU stays. We also evaluated the impact of using the two GFR measurements on the estimation of complete recovery relative to baseline eGFR. Parameters were compared with the paired *t *test and McNemar's test.

## Results

Amongst the 784 patients with AKI, 456 (58%) reached stage 1, 143 (18%) stage 2 and 185 (24%) stage 3. Mean ± SD Clcr and eGFR at ICU discharge were respectively 54.5 ± 28 and 76 ± 55 ml/minute/1.73 m^2 ^(*P *< 0.0001). eGFR was not significantly different from Clcr in patients with ICU stay <7 days. In patients with ICU stay between 8 and 14 days, eGFR was significantly higher than Clcr (79 ± 51 vs. 48.5 ± 20, *P *< 0.0001) and the difference increased even further in patients with ICU stay over 14 days (102 ± 70 vs. 42.6 ± 20, *P *< 0.0001). The percentage of patients with complete recovery differed significantly when evaluated by eGFR (35.3%) or Clcr (28.7%) (*P *= 0.007). In patients with ICU stay >14 days, this difference increased to 56.4% by eGFR versus 14.1% by Clcr (*P *< 0.0001).

## Conclusion

Estimated GFR at ICU discharge is significantly higher than the measured Clcr in patients with prolonged ICU stay. This difference can be explained by loss of muscle mass with decreased creatinine production and results in an important overestimation of recovery.
